# Action Recognition of Taekwondo Unit Actions Using Action Images Constructed with Time-Warped Motion Profiles

**DOI:** 10.3390/s24082595

**Published:** 2024-04-18

**Authors:** Junghwan Lim, Chenglong Luo, Seunghun Lee, Young Eun Song, Hoeryong Jung

**Affiliations:** 1 Department of Motion, Torooc Co., Ltd., Seoul 04585, Republic of Korea; gpopl123@naver.com; 2 Department of Mechanical Engineering, Konkuk University, Seoul 05029, Republic of Korea; luo0611@konkuk.ac.kr; 3 School of Mechanical and Aerospace Engineering, Seoul National University, Seoul 08826, Republic of Korea; erioer95@snu.ac.kr; 4 Department of Autonomous Mobility, Korea University, Sejong 30019, Republic of Korea

**Keywords:** action recognition, convolution neural network, human action dataset, taekwondo

## Abstract

Taekwondo has evolved from a traditional martial art into an official Olympic sport. This study introduces a novel action recognition model tailored for Taekwondo unit actions, utilizing joint-motion data acquired via wearable inertial measurement unit (IMU) sensors. The utilization of IMU sensor-measured motion data facilitates the capture of the intricate and rapid movements characteristic of Taekwondo techniques. The model, underpinned by a conventional convolutional neural network (CNN)-based image classification framework, synthesizes action images to represent individual Taekwondo unit actions. These action images are generated by mapping joint-motion profiles onto the RGB color space, thus encapsulating the motion dynamics of a single unit action within a solitary image. To further refine the representation of rapid movements within these images, a time-warping technique was applied, adjusting motion profiles in relation to the velocity of the action. The effectiveness of the proposed model was assessed using a dataset compiled from 40 Taekwondo experts, yielding remarkable outcomes: an accuracy of 0.998, a precision of 0.983, a recall of 0.982, and an F1 score of 0.982. These results underscore this time-warping technique’s contribution to enhancing feature representation, as well as the proposed method’s scalability and effectiveness in recognizing Taekwondo unit actions.

## 1. Introduction

Taekwondo, originating from Korea, has evolved from a traditional martial art into an official Olympic sport, becoming one of the world’s most practiced martial arts. This discipline is divided into two main categories: Gyeorugi, a full-contact sparring between two competitors utilizing electronic scoring equipment for objective, quantitative assessments, and Poomsae, where individual competitors perform a series of predetermined movements, including basic attack and defense techniques, in front of judges. The evaluation of Poomsae is inherently subjective and qualitative, lacking objective tools for assessment, which complicates the recognition of competitors’ movements and introduces fairness concerns due to variability in judges’ assessments.

The application of action recognition technology, which enables precise motion measurement and quantitative evaluation, presents a promising solution to the challenges faced in current Poomsae evaluations [1,2,3,4,5,6,7,8,9,10,11]. A variety of vision-based action recognition methods have been developed specifically for Taekwondo. De Goma et al. [12] utilized a hidden Markov model (HMM) with skeletons extracted from RGB-D camera images for action recognition. Choi et al. [13] introduced a remote evaluation module for Poomsae using a multi-view sensor action recognition approach. Seo et al. [14] developed a recognition algorithm based on Poisson distribution, leveraging one-dimensional spatial information from image sequences. Kong et al. [15] proposed an automatic analysis framework for broadcasted Taekwondo videos, integrating a structure-preserving object tracker with a principal component analysis (PCA) network. Liang et al. [16] explored a novel evaluation method for Taekwondo competitions combining long short-term memory (LSTM) with a spatial temporal graph convolutional network (ST-GCN). Recently, Lee et al. [17] reported over 80% recognition accuracy using a convolutional neural network (CNN)-based model that processes a sequence of key-frame images to identify basic Taekwondo unit actions. However, these vision-based strategies, relying on raw image sequences, face significant accuracy limitations due to environmental conditions and the appearance of competitors’ attire.

Skeleton-based action recognition models represent a promising avenue for mitigating the limitations encountered in traditional vision-based action recognition, particularly by excluding extraneous background elements that are not pertinent to recognizing actions [18,19,20,21]. This methodology hinges on extracting the subject’s skeleton from the image, which is then utilized as the input for the action classification model. By eliminating superfluous details from the raw images, this approach ensures that accuracy is not compromised by environmental variables. Recent studies have been exploring the application of conventional CNN-based methodologies to vision-based action recognition using extracted skeletons [22,23,24,25,26,27]. Muhammad et al. propose a hybrid two-stream convolutional neural network (G2SCNN) for recognizing actions in sequences to identify structured patterns of body parts and movements [24]. The G2SCNN demonstrated 87.28% performance accuracy on benchmark datasets. Zan et al. suggest a long-short term memory model combining CNNs and LSTM networks (TS-CNN-LSTM) to overcome the long duration and low-latency issues inherent in traditional human action recognition [25]. This model achieved a maximum accuracy of 87.28% in cross-subject assessments, while its temporal complexity occupies only 67.5% and its spatial complexity occupies 73.98%. Yue Ming et al. attempt to alleviate accuracy degradation due to the lack of local detailed information in RGB-based transformers by proposing a frequency-spatial-domain CNN transformer of a two-stream network (FSConformer) for compressed video action recognition [26]. FSConformer reached a higher accuracy compared to other compressed-domain methods on UCF-101, Kinetics-400, and Kinetics-700 datasets. Malik et al. developed a high-accuracy multi-view action recognition system using 2D skeleton data. By combining OpenPose and CNN-LSTM architecture, they achieved an accuracy of 94.4% on the MCAD (multi-camera action dataset) [27].

Graph convolutional network (GCN)-based strategies [28], which employ action classifiers trained on input graphs derived from skeletal configurations, along with 3D convolutional networks that utilize 3D heat maps of the skeletons [29], have been introduced to enhance the robustness of action recognition. These techniques enable the action classifier to discern the essential characteristics of an action through the geometric data of a joint and its adjacent points, independent of background elements. However, a significant challenge arises owing to their sensitivity to minor coordinate alterations, which can result in markedly divergent predictions [30,31,32]. This issue is particularly pronounced in martial arts like Taekwondo, where rapid and intricate movements may lead to inaccuracies in joint positioning and topological errors, consequently affecting the stability and consistency of action predictions [33,34].

Despite the predominance of vision-based methodologies in action recognition research, they exhibit several drawbacks when applied to Taekwondo action recognition. An alternative approach that circumvents these limitations involves the use of wearable inertial measurement unit (IMU) sensors. These sensors, affixed to the body’s joints, capture three-dimensional linear accelerations and angular velocities, facilitating the computation of full-body motion in a rapid (>200 Hz) and reliable manner. Prior research has explored the application of various machine learning techniques to process raw IMU data for action classification, including recurrent neural networks (RNNs) [35], LSTM networks [36], CNNs [37], and hybrid CNN-LSTM models [38], showcasing the potential of IMU sensors in overcoming the challenges posed by vision-based action recognition methods.

Recent investigations have explored the conversion of raw IMU data into the RGB color space to generate action images, which are subsequently utilized as inputs for action classification [39]. This advanced technique captures both the spatial attributes of human motion—such as joint positions, velocities, and accelerations—and the temporal dynamics by integrating sequential action data within a defined time frame into a singular image, thereby offering a comprehensive input for the action classifier. Despite the potential of this approach, the specific inclusion and representation of action characteristics within the action image have been inadequately addressed.

In this study, we introduce an action recognition model specifically designed for Taekwondo unit actions, utilizing motion data obtained from wearable IMU sensors. The proposed method refines the existing strategy of transforming joint-motion data into RGB action images by applying a modulation of the time-domain motion profiles. This modification aims to amplify the depiction of rapid movements characteristic of Taekwondo unit actions. The main concept of our approach is the implementation of time-warping techniques to the motion profiles based on their velocity, thereby extending the portrayal of swift movements within the action image. A CNN-based classification model was adopted to evaluate the effectiveness of our proposed method, employing a dataset of Taekwondo unit actions from 40 skilled practitioners. The primary contributions of our study are outlined as follows:

1. We present a pioneering technique for creating an action image from joint-motion data captured via IMU sensors. This method incorporates time-warping techniques into the motion profiles, significantly enhancing the representation of rapid movements within the action image.
2. The effectiveness of the proposed action recognition model was validated with a dataset comprising Taekwondo unit actions from 40 experts. The evaluation results not only affirm the model’s accuracy but also its scalability, underscoring the viability of our approach in recognizing Taekwondo unit actions.

## 2. Materials and Methods

**Figure 1:**
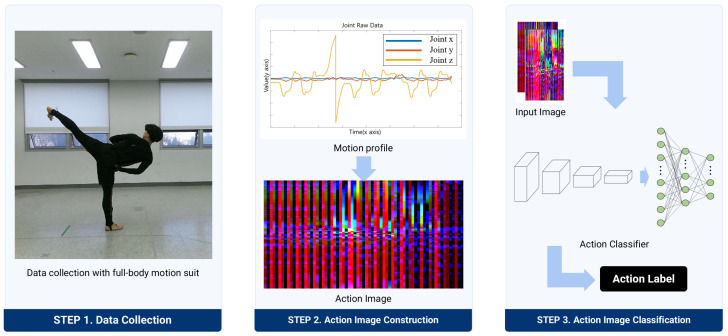
Overview of the proposed action recognition process.

The action recognition framework proposed in this study is structured around three principal phases: data collection, action image generation, and action classification, as depicted in Figure 1.

Data collection: This phase involves the acquisition of IMU data from sensors affixed to participants as they execute specific Taekwondo unit actions. The gathered data are segmented into individual actions and annotated with the corresponding action names to facilitate subsequent analysis.

Action image generation: In this step, the segmented IMU data for each unit action are transformed into a singular action image. This transformation is achieved by mapping the IMU data values to the RGB color spectrum of each pixel, with the image’s columns representing the sampling times and the rows reflecting the indices of the IMU sensors.

Action classification: The final phase employs a CNN classification model to discern the Taekwondo unit actions from the generated action images. This model is specifically designed to process action images derived from IMU data, outputting the designated label for each action.

### 2.1. Data Collection

During this study, a dataset comprising IMU data on Poomsae unit actions was compiled, drawing from the expertise of professional Taekwondo practitioners. The data collection protocol received approval from the Konkuk University Institutional Review Board (IRB) under protocol number 7001355-202004-HR-372, ensuring the process adhered to ethical guidelines. The dataset was devoid of any personally identifiable information, and informed consent was secured from each participant prior to data collection. The participants were assured that the data would be exclusively utilized for scholarly research purposes.

#### 2.1.1. Participants

For the data collection endeavor, forty adult professional Taekwondo Poomsae demonstrators were enlisted. These participants were assigned identifiers ranging from T1 to T40, enabling the systematic categorization of the data derived from different individuals. The participants had an age of 21.38 ± 3.57 years, a height of 173.50 ± 4.82 cm, and a weight of 69.27 ± 7.61 kg.

#### 2.1.2. Data Collection Protocol

**Figure 2:**
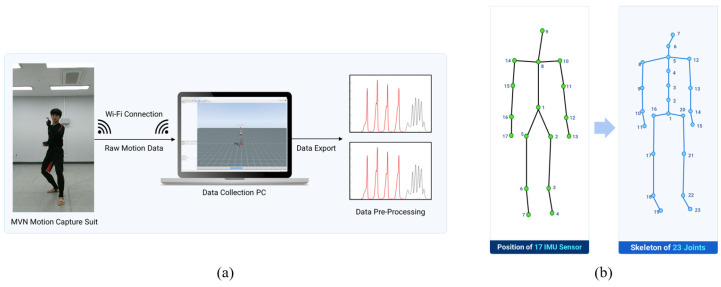
Data collection setup. (**a**) Configuration of the data collection setup. (**b**) Placement of IMU sensors and the skeleton structure, illustrating 23 joints.

**Figure 3:**
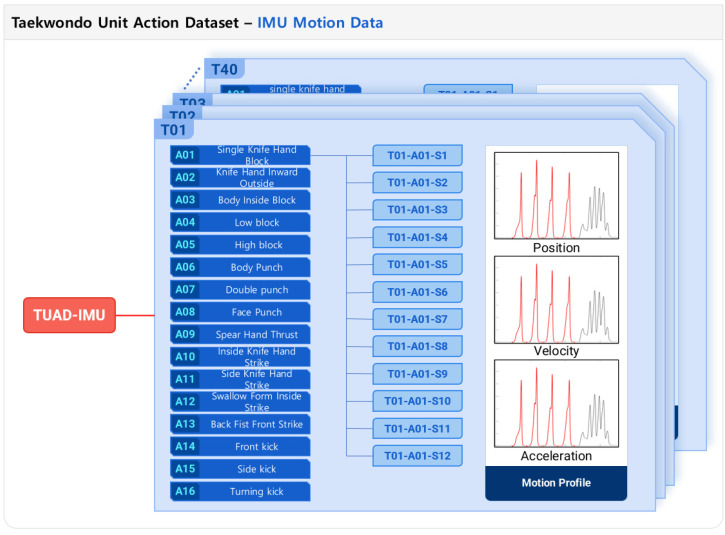
Structure of the Taekwondo unit action dataset (TUAD-IMU) derived from the IMU motion data.

Figure 2a illustrates the configuration utilized for data collection. Participants were equipped with a comprehensive motion capture ensemble, specifically the Xsens MVN system (Xsens Corp., Enschede, The Netherlands). This ensemble comprised a snugly fitting Lycra suit integrated with 17 IMU sensors designated for motion tracking (MTx, MVN, Xsens Corp., The Netherlands), accompanied by a waist pack housing the batteries, a data acquisition (DAQ) unit, and a wireless transmission module facilitating data exchange with the central computing unit. The system afforded the capability of real-time, full-body motion tracking, capturing data across 23 joints. These included nine sets of translational data (encompassing position, velocity, and acceleration) and nine sets of angular data (covering orientation, angular velocity, and angular acceleration), all sampled at a frequency of 240 Hz. These measurements were derived from the 17 IMU sensors through sophisticated, embedded motion-tracking algorithms. For an in-depth explanation, reference [40] provides further details. The placement of the sensors and corresponding joints is depicted in Figure 2b. During the data collection phase, participants were instructed to sequentially perform 16 distinct Poomsae unit actions while donning the motion capture suit. Table 1 delineates the names and descriptions of the sixteen unit actions performed by the experts. Each unit action was performed four times in succession before the participant reverted to their starting position. This routine was replicated three times for each unit action, culminating in 12 iterations per action. Through this meticulous procedure, a total of 7680 datasets of Taekwondo unit actions were amassed (calculated from 40 participants, each performing 16 unit actions, repeated 12 times), as depicted in Figure 3.

### 2.2. Action Image Generation

#### 2.2.1. Action Segmentation

**Figure 4:**
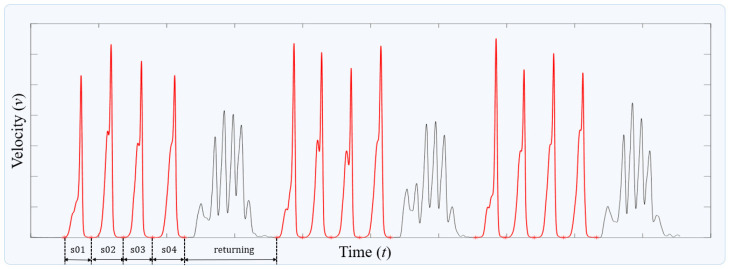
Process of segmenting a single action profile. The red lines represents the segmented unit-action profiles.

During the data acquisition phase, subjects were required to execute a specific unit action 12 times in succession. Given that each action image corresponds to a singular unit action, the IMU data, encompassing 12 repetitions of the unit action, needed to be segmented into 12 distinct datasets. This process employed the average velocity value—calculated by averaging the magnitudes of velocities recorded at the hands and feet—as a criterion to determine the initial and final samples of each action, as depicted in Figure 4. This velocity metric facilitated the straightforward segmentation of the motion profile according to individual unit actions, as illustrated in the figure.

**Table 1:** Description of 16 unit actions performed by the Taekwondo experts.

ID	Name	Description
A1	Single Knife-Hand Block	A defensive movement using the side of the hand to block an attack.
A2	Knife-Hand Inward Outside	A knife-hand technique moving from inward to outward to block or strike.
A3	Body Inside Block	A block executed with the arm moving inward to protect the body from an incoming strike.
A4	Low Block	A block aimed downward to defend against low strikes to the body.
A5	High Block	A block aimed upward to protect the head and upper body from high strikes.
A6	Body Punch	A punch directed toward the body of the opponent.
A7	Double Punch	Two consecutive punches usually aimed at the body or head with one hand following the other.
A8	Face Punch	A punch aimed directly at the opponent’s face.
A9	Spear-Hand Thrust	A straight thrust with the hand shaped like a spear, targeting vital spots.
A10	Inside Knife-Hand Strike	A strike using the side of the hand, moving from inward to outward toward the opponent.
A11	Side Knife-Hand Strike	A sideways strike with the knife hand, targeting the opponent’s neck or ribs.
A12	Swallow-Form Inside Strike	A complex, flowing strike that mimics the movement of a swallow, executed inside to outside.
A13	Back-Fist Front Strike	A quick strike using the back of the fist, directed toward the opponent’s front.
A14	Front Kick	A straightforward kick aimed at the opponent, which can target the opponent’s body or head.
A15	Side Kick	A kick executed sideways, powerful for attacking the opponent’s side or breaking their guard.
A16	Turning Kick	A kick involving a turning motion, increasing the power through momentum.

#### 2.2.2. Projecting IMU Data onto the RGB Color Space

**Figure 5:**
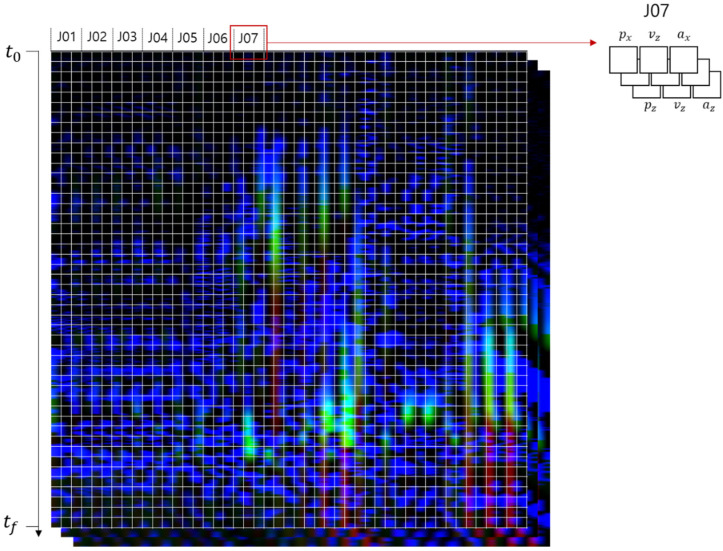
Generation of action images through mapping the IMU motion data onto the RGB color space.

The methodology for generating an action image involved mapping the IMU data values onto the RGB color space of image pixels. This process transformed the time-series data of both linear and angular motions across 23 joints, associated with a single unit action, into one comprehensive action image. The linear and angular motion data included 3-dimensional aspects of position, velocity, and acceleration, culminating in 18 scalar values for each joint per time frame. Figure 5 elucidates the structure of the action images, wherein the column in the action image signifies the joint index and the type of motion data. The six motion data points were represented by six columns within the action image, with the x, y, and z dimensions of each motion data point corresponding to the R, G, and B color channels of the image pixels, respectively. For instance, the motion data for joint 1 (J01) would occupy the first six columns of the image, with the 3-dimensional space of the motion data being mapped onto the three channels of the image pixel.

In the process of mapping the sampling time to pixel row coordinates and the joint index to pixel column coordinates, the RGB values of the image pixels were derived from the IMU data as per the following methodology:

(1) $$C_{i,j}^{red}=\frac{1}{{P_{x}(j)}^{max}-{P_{x}(j)}^{min}}{P_{x}(j)}_{i},$$

(2) $$C_{i,j}^{green}=\frac{1}{{P_{x}(j)}^{max}-{P_{x}(j)}^{min}}{P_{y}(j)}_{i},$$

(3) $$C_{i,j}^{blue}=\frac{1}{{P_{x}(j)}^{max}-{P_{x}(j)}^{min}}{P_{z}(j)}_{i},$$

where $C_{i,j}^{red}$ represents the pixel value of the red channel at the *i*-th row and *j*-th column, and P(j) represents the function that returns the motion data corresponding to the *j*-th column of the image at the *i*-th time frame. ${P(j)}^{max}$ and ${P(j)}^{min}$ denote the maximum and minimum values of P(j), respectively.

#### 2.2.3. Time Warping

The rapid movements inherent in Taekwondo unit actions present a significant challenge for action recognition. As these dynamic movements are pivotal in distinguishing Taekwondo actions, their representation in action images necessitates emphasis. In the described action image construction, each row correlates to a distinct time frame of an action, distributing the temporal sequence of the action uniformly across the rows, irrespective of the action’s velocity. This uniform distribution results in a diminished representation of rapid movements compared to slower ones. Despite the critical role of rapid movements in the recognition of Taekwondo actions, their depiction in action images has been insufficiently accentuated.

To address this issue, this study introduces a time-warping technique designed to enhance the depiction of rapid movements in action images. The concept of time warping mirrors the slow-motion effects utilized in cinematography, which decelerates the footage to capture and emphasize essential or fleeting details without missing them. By expanding the temporal segments corresponding to rapid movements within an action image, this technique aims to facilitate a more effective capture of the fundamental characteristics of Taekwondo actions. The application of this time-warping method employs a velocity indicator—calculated by averaging the velocity magnitudes of both the hands and feet—to delineate the speed of motion, thereby adjusting the representation of rapid movements within the action image to ensure they are adequately emphasized.

(4) $$V^{rep}(t)=\frac{1}{4}(\left\|V^{hl}(t)\right\|+\left\|V^{hr}(t)\right\|+\left\|V^{fl}(t)\right\|+\left\|V^{fr}(t)\right\|),$$

where $V^{rep}(t)$ represents the representative speed of motion at time *t*, and $V^{hl}(t)$, $V^{hr}(t)$, $V^{fl}(t)$, and $V^{fr}(t)$ denote the velocities of the hands and feet in the corresponding time frame. We assume that the motion data of one unit of action are presented as a function of time *t*, as follows:

(5) $$p=p\left(t\right),\;where\;0<t<t_{f},\;$$

where $t_{f}$ denotes the final time of the action. The time-warping algorithm modifies the time domain to accentuate segments corresponding to rapid movements, thereby transforming the original time domain into a warped time domain using the value of $V^{rep}$ as follows:

(6) $$t^{warp}=\frac{\int_{0}^{t}V^{rep}\left(t\right)dt}{\int_{0}^{t_{f}}V^{rep}\left(t\right)dt}\times t_{f}.$$

Upon substituting the time *t* into the proposed equation, the corresponding warped time $t^{warp}$ is computed, allowing for the determination of position data within this altered time frame using the following expression:

(7) $$p^{warp}=p\left(t^{warp}\right).$$

These position data, once resampled, serve as the basis for generating the action image utilized in the classification process. The detailed procedure of the time-warping algorithm is presented in the following pseudo code (Algorithm 1).
**Algorithm 1** Time Warping of Motion Profile**function** TIME_WARPING (P, $V^{rep}$, $P^{warp}$)     **Input**   P: motion profile that should be modified by the time-warping algorithm        $V^{rep}$: representative velocity profile     **Output** $P^{warp}$: time-warped motion profile1:       $t_{f}$ = *Size*(P)                   % counts the number of samples in motion profile P
2:       TotalIntegral = 0            % initialize the variable calculating the total sum of $V^{rep}$
3:       **for** $t=1\;:\;t_{f}$
4:           TotalIntegral += $V^{rep}[t]$
5:       **end for**6:       PartialIntegral = 0         % initialize the variable calculating the partial sum of $V^{rep}$
7:       **for** $t=1\;:\;t_{f}$
8:             PartialIntegral+=$V^{rep}[t]$
9:             $t^{warp}$ = PartialIntegral/TotalIntegral × *t*10:            $P^{warp}[t]$ = *Spline*(P, $t^{warp}$)    % spline interpolation at $t^{warp}$
11:        **end for**

### 2.3. CNN Architecture

**Figure 6:**
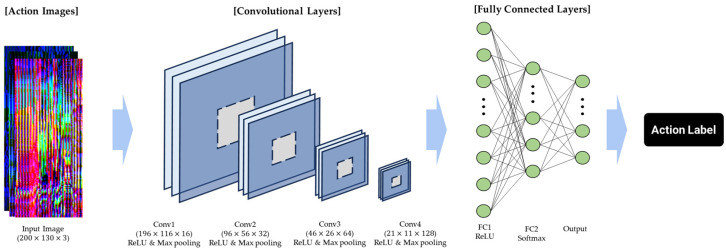
Architectural diagram of the CNN used for action classification.

The CNN-based classification model, which employs the generated action image as its input, is tasked with identifying the appropriate action label. The model’s architecture, as depicted in Figure 6, encompasses four convolutional layers followed by fully connected layers. The convolutional layers are equipped with filters of dimensions 5 × 5 × 16, 3 × 3 × 32, 3 × 3 × 64, and 3 × 3 × 128, respectively. The rectified linear unit (ReLU) functions as the activation mechanism, while max pooling is implemented with 2 × 2 windows and a stride of 2 to reduce spatial dimensions. Table 2 presents the output shapes and parameter counts for each layer within the model. For the training and validation of the model, 70% of the action images were allocated for training, with the remaining 30% being dedicated to validation. To ensure thorough training and validation, a 5-fold cross-validation technique was employed, enhancing the robustness and reliability of the classification outcomes.

### 2.4. Evaluation Metrics

The effectiveness of the action recognition model presented in this study was assessed utilizing four key metrics: accuracy, precision, recall, and F1 score. These metrics were calculated as follows:

(8) $$accuracy=\frac{TP+TN}{TP+TN+FP+FN},precision=\frac{TP}{TP+FP},\;recall=\frac{TP}{TP+FN},F1score=2\cdot \frac{precision\cdot recall}{precision+recall}$$

where TP, TN, FP, and FN represent true positives, true negatives, false positives, and false negatives, respectively.

## 3. Results

The validation of the proposed action recognition model was conducted through a dual approach. Initially, a performance evaluation was carried out to compare the models that utilized action images generated from time-warped motion data against those created from standard motion data. The objective was to ascertain the effectiveness of the time-warping algorithm. Subsequently, the model’s performance was examined in relation to the number of joints considered for generating the action image, aiming to assess the algorithm’s scalability. For this comparison, action images were generated using motion data from varying numbers of joints: 23, 8, and 4.

### 3.1. Performance Evaluation for the Time-Warped Action Images

**Figure 7:**
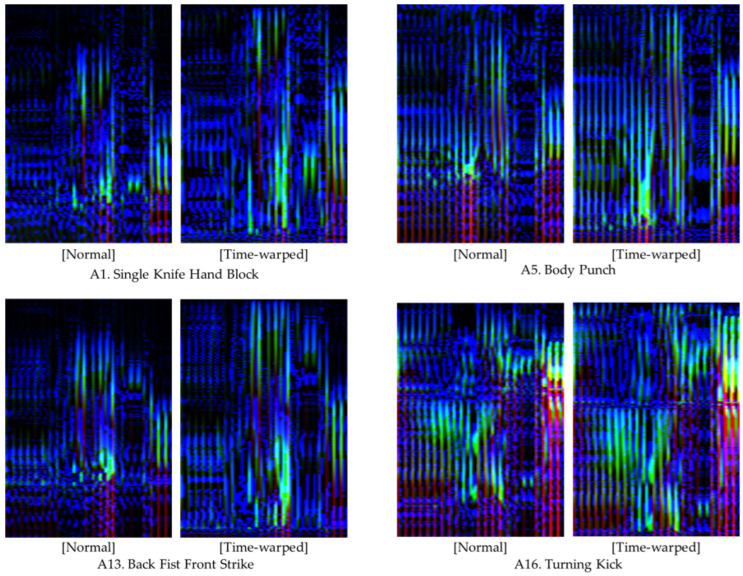
Comparative illustrations of action images generated from normal and time-warped motion data.

Figure 7 displays the action images for four distinct unit actions, created using both time-warped and normal motion data. The color intensity within these images, with brighter colors indicating higher data magnitudes, showcase that the application of time-warping techniques resulted in an expanded bright region. This expansion signifies an enhancement in the representation of rapid motion features within the action images. Table 3 delineates the results of the performance comparison across the 16 unit actions using the four evaluation metrics, with approximately 140 action images per unit action employed for model performance evaluation.

The comparative analysis revealed that both methodologies exhibited high levels of accuracy and precision across all evaluated actions. For example, in the case of Action1, the “time-warping” technique achieved an accuracy of 0.999 and a precision of 1.000, marginally surpassing the “normal” method, which recorded an accuracy of 0.997 and a precision of 0.993. This pattern of performance enhancement through the “time-warping” technique was consistent across the majority of actions. Actions such as Action2 and Action14 were particularly noteworthy, where the “time-warping” approach attained perfect scores (1.000) in both metrics, underscoring its effectiveness. The exploration of recall and F1 scores unveils subtle distinctions between the “normal” and “time-warping” methods. For instance, in Action3, the “normal” method yielded a recall of 0.977 and an F1 score of 0.927. In contrast, the “time-warping” method exhibited a slightly lower recall of 0.921 yet achieved a superior F1 score of 0.942. Such discrepancies suggest that while the “time-warping” method may occasionally compromise recall for precision, it broadly sustains a high level of performance, as reflected by its consistently elevated F1 scores.

**Figure 8:**
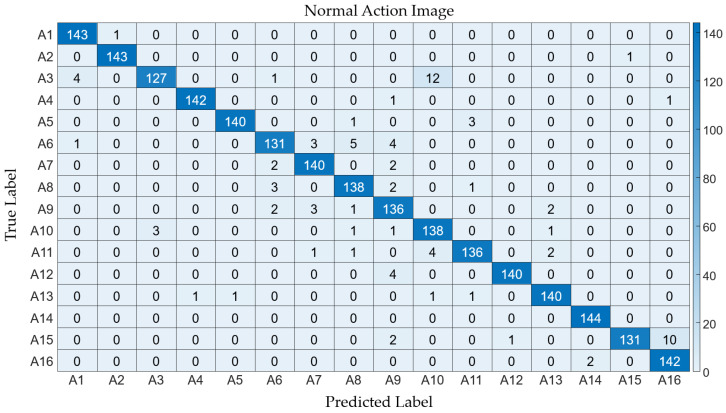
Confusion matrix illustrating the classification results from the normal action images.

**Figure 9:**
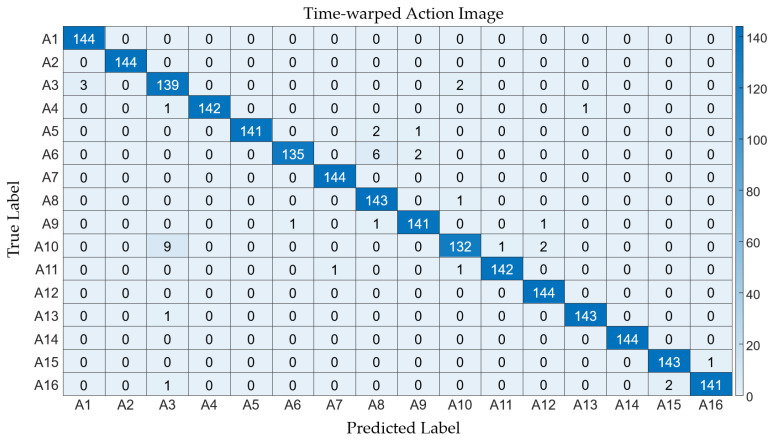
Confusion matrix illustrating the classification results from the time-warped action images.

Collectively, the time-warping method demonstrated marginally superior efficacy across the board. The average performance metrics for the “time-warping” method—accuracy: 0.998, precision: 0.982, recall: 0.982, F1 score: 0.982—surpassed those of the “normal” method—accuracy: 0.995, precision: 0.960, recall: 0.960, F1 score: 0.960. This denotes a slight yet consistent edge of the “time-warping” approach in action recognition endeavors, producing detailed classification outcomes, as presented in Figure 8 and Figure 9.

### 3.2. Performance Evaluation According to the Number of Joints

**Figure 10:**
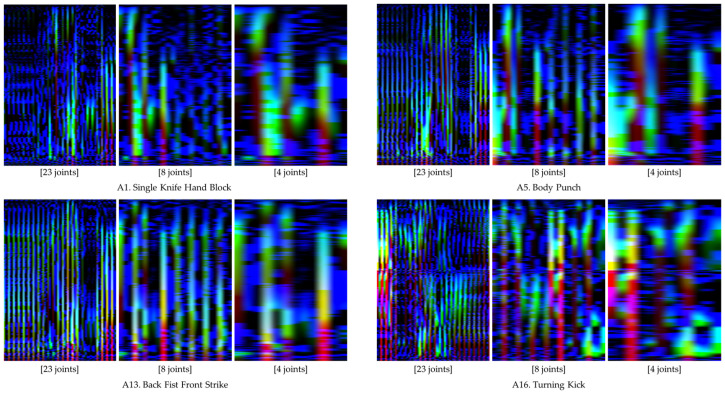
Comparative display of action images generated using motion data from 23, 8, and 4 joints.

**Figure 11:**
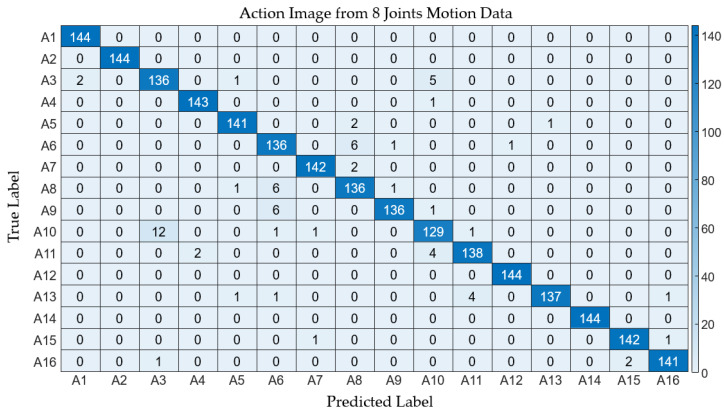
Confusion matrix depicting the classification results derived from motion data involving 8 joints.

**Figure 12:**
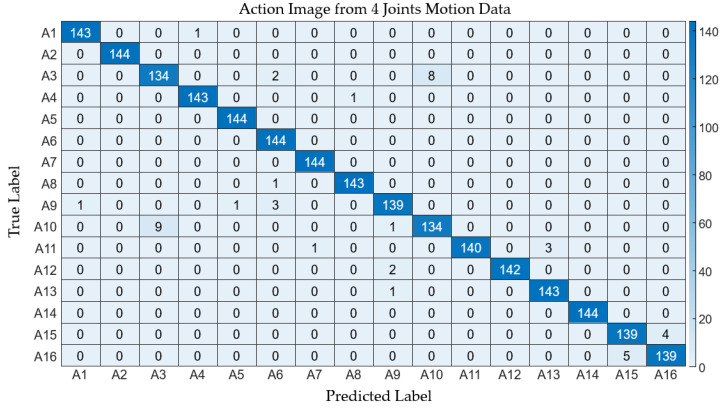
Confusion matrix depicting the classification results derived from motion data involving 4 joints.

In the context of employing wearable IMU sensors for action recognition, reducing the number of sensors is imperative to curtail both costs and system complexity. This segment of the study delves into the scalability of the proposed method in relation to the number of joints considered for generating an action image. To evaluate performance, we employed three variants of action images, derived from the time-warped motion data of 23, 8, and 4 joints, respectively. Figure 10 delineates these action image types. The intrinsic features of the action images are effectively conveyed in both the comprehensive (23 joints) and the condensed (8 and 4 joints) formats, as depicted in the figure. Table 4 enumerates the performance metrics across the three action image scales. It can be observed that the average values of the performance metrics exhibit a slight decrement with the reduction in the number of joints involved. Specifically, the average accuracy for the comprehensive action images stood at 0.998, while the reduced-scale images registered average accuracies of 0.998 and 0.996 for eight and four joints, respectively, with analogous patterns being observable in other metrics. The detailed classification outcomes are showcased in Figure 11 and Figure 12.

### 3.3. Performance Comparison with Previous Models

The performance of the proposed model was compared with the previous action recognition models. In this performance comparison, the Taekwondo unit action dataset collected in this research was used for both training and testing the previous models. Table 5 presents a performance comparison of the proposed model with the previous models. The proposed model demonstrated superior performance over the previous models across all evaluation metrics.

### 3.4. Sensitivity Analysis through Input Data Perturbation

We evaluated the sensitivity of the proposed model by examining its performance under varying conditions of the input data. To simulate realistic scenarios where the data might be subject to measurement errors or external perturbations, we intentionally introduced errors into the motion profiles derived from the IMU sensors. These errors were modeled to have a normal distribution and were superimposed onto the mean values of the respective datasets. The magnitude of these artificial errors was carefully chosen to represent minor (1%) and more noticeable (3%) deviations from the original data. By creating these two distinct datasets, we aimed to mimic potential inaccuracies that could arise in real-world applications and observe how such variations could influence the predictive capabilities of our model. Table 6 shows the results of the sensitivity analysis. The action recognition performance tended to degrade as the errors in the input data increased, but even with a 3% error rate in the input data, it demonstrated a reliable level of accuracy.

## 4. Discussion

This study presents an innovative action recognition model designed for the quantitative evaluation of Poomsae Taekwondo using action images derived from motion data collected via wearable IMU sensors. This approach successfully addresses a critical shortcoming of previous vision-based methodologies, namely the difficulty in accurately capturing features indicative of rapid movements. By integrating rapidly updated IMU data with a time-warping modulation, the model effectively adjusts motion profiles within the time domain based on velocity, thereby significantly enhancing the depiction of rapid movements. Employing a CNN-based classification model to implement this method has showcased substantial efficacy across various metrics, underlining the model’s robustness and potential applicability in the precise assessment of Poomsae performances.

The findings from the performance comparison substantiate that the time-warping method significantly augments action recognition capabilities. Through the application of time warping, there were marked improvements in the average metrics of accuracy, precision, recall, and F1 score by 0.30%, 2.28%, 2.29%, and 2.29%, respectively. This method is particularly effective in enhancing the differentiation of actions that exhibit closely related motion characteristics. For example, the unit actions A6 (body punch) and A8 (face punch) demonstrate nearly indistinguishable motions, with the primary variance being the hand’s direction during the punch. The rapid motion profile of the “punch” is more accurately represented through the application of time warping, leading to an improvement in performance. Specifically, the accuracy for A6 (body punch) increased from 99.1% to 99.6%, and for A8 (face punch), it rose from 99.3% to 99.6%. These enhancements underscore the efficacy of time warping in classifying motions with subtle differences, validating its effectiveness in the complex domain of martial arts technique recognition.

The utility of time warping extends to unit actions involving kicks, such as A14 (front kick), A15 (side kick), and A16 (turning kick), where it facilitated notable improvements across all evaluated performance metrics. For A14 (front kick), the metrics of accuracy, precision, recall, and F1 score improved from 99.9% to a perfect 100%. A15 (side kick) saw increases in accuracy (from 99.4% to 99.9%), precision (from 98.5% to 99.6%), recall (from 88.7% to 99.8%), and F1 score (from 93.8% to 99.6%). Similarly, A16 (turning kick) experienced enhancements in accuracy (from 99.4% to 99.8%), precision (from 93.4% to 99.6%), recall (from 92.8% to 99.2%), and F1 score (from 98.6% to 99.6%). These advancements in the recognition of Taekwondo kicks, especially where action profiles may share similar final poses but differ in movement trajectories and speeds, highlight the profound impact of time warping. It proves particularly beneficial in distinguishing between similar kick actions, thereby confirming its substantial value in the nuanced recognition of Taekwondo techniques.

In assessing the practical implications of the proposed action recognition model, this study delved into the system’s scalability relative to the quantity of joint-motion data utilized. A key advantage of reducing the number of sensors is the consequent decrease in both the cost and complexity of the equipment required, thereby rendering the technology more accessible and user-friendly. Crucially, the findings from this investigation reveal that the performance of the action recognition system remains robust, even when the amount of joint-motion data is significantly reduced. This outcome is of paramount importance, demonstrating that a streamlined sensor setup, when integrated with the time-warping technique, is capable of maintaining high levels of accuracy and efficiency. Such insights are invaluable for practical implementations where the objectives include minimizing costs and simplifying operational complexity without sacrificing the accuracy of the system.

Considering the real-world application of the proposed model, there are certain limitations that may affect its practicality. Firstly, the requirement for competitors to wear device-equipped attire introduces a level of complexity in the experimental setup and could potentially hinder natural movement due to discomfort or unfamiliarity with the wearable devices. Moreover, while our model has been validated with datasets obtained from Taekwondo experts, its accuracy may diminish when applied to non-experts due to the variations in motion execution. In terms of computational efficiency, the computational burden of the proposed model is linearly dependent on the number of parameters in the classification model (921,845), which may pose challenges for real-time processing in the embedded processors of the wearable devices. Furthermore, the necessity to segment continuous motion in real time introduces additional computational demands. Addressing these aspects is crucial for enhancing the model’s practicality and effectiveness in real-world scenarios.

The focus of the current research has been on the classification of the 16 individual unit actions characteristic of Taekwondo. Future research should endeavor to extend beyond this scope to encompass the evaluation of Poomsae. Poomsae represents sequences that combine various unit actions, wherein the precision and fluidity of each constituent movement are of critical importance. Drawing upon the knowledge acquired from the successful recognition and analysis of individual unit actions, advancing the development of algorithms capable of evaluating and analyzing the comprehensive execution of Poomsae emerges as a vital subsequent step. Moreover, collecting additional data from both experts and non-experts across different demographics is necessary to apply the proposed model in real-world scenarios. This progression will not only enhance the understanding and assessment of Poomsae performances but also contribute significantly to the broader field of motion analysis, such as in sports, physical rehabilitation, and healthcare applications. Within the field of sports, precise and efficient action recognition capabilities could contribute to analyzing and improving athletes’ techniques, offering real-time feedback that could enhance their performance and reduce the risk of injury. This would be particularly beneficial in sports requiring precise movements and coordination, such as gymnastics, swimming, and athletics. As this discussion underscores, the potential applications and implications of the proposed action recognition model are vast and varied. Future research will need to focus on tailoring the model to specific applications, improving its accuracy and adaptability, and exploring ways to integrate it into existing systems and technologies.

## 5. Conclusions

This paper presents an action recognition model tailored for Taekwondo unit actions, employing action images generated from full-body joint-motion data captured via IMU sensors. The proposed model augments the representation of rapid motion by modulating motion profiles through the application of time-warping techniques, thereby facilitating the identification of subtle differences in motion characteristics. A comparative analysis of its performance underscored not only this method’s efficacy but also its scalability, affirming its utility across varying scales of joint-motion data. In conclusion, our research contributes a novel approach to the recognition and analysis of Taekwondo actions, underscoring the potential of wearable IMU sensors in capturing the nuances of fast and complex movements. This advancement marks a significant step forward in the integration of technology with traditional sports training and assessment practices, heralding a future where technological innovations enhance athletic performance and training methodologies.

## Figures and Tables

**Table 2:** Specifications of the classification model including the output shapes and parameter counts.

Layer Index	Conv1	Conv2	Conv3	Conv4	FC1	FC2	Total
Output shape	196 × 116 × 16	96 × 58 × 16	46 × 26 × 64	23 × 13 × 64	6400 × 1	128 × 1	-
The number of parameters	2416	4640	18,496	73,856	819,328	4128	921,854

**Table 3:** Comparative analysis of performance metrics between the time-warped and normal action images.

	Accuracy		Precision		Recall		F1 Score	
	Normal	Warped	Normal	Warped	Normal	Warped	Normal	Warped
A1	0.997	0.999	0.966	0.980	0.993	1.000	0.979	0.990
A2	0.999	1.000	0.993	1.000	0.993	1.000	0.993	1.000
A3	0.991	0.993	0.977	0.921	0.882	0.965	0.927	0.942
A4	0.999	0.999	0.993	1.000	0.986	0.986	0.990	0.993
A5	0.998	0.999	0.993	1.000	0.972	0.979	0.982	0.989
A6	0.991	0.996	0.942	0.993	0.910	0.938	0.926	0.964
A7	0.995	1.000	0.952	0.993	0.972	1.000	0.962	0.997
A8	0.993	0.996	0.939	0.941	0.958	0.993	0.948	0.966
A9	0.990	0.997	0.895	0.979	0.944	0.979	0.919	0.979
A10	0.990	0.993	0.890	0.971	0.958	0.917	0.923	0.943
A11	0.994	0.999	0.965	0.993	0.944	0.986	0.954	0.990
A12	0.998	0.999	0.993	0.980	0.972	1.000	0.982	0.990
A13	0.996	0.999	0.966	0.993	0.972	0.993	0.969	0.993
A14	0.999	1.000	0.986	1.000	1.000	1.000	0.993	1.000
A15	0.994	0.999	0.992	0.986	0.910	0.993	0.949	0.990
A16	0.994	0.998	0.928	0.993	0.986	0.979	0.956	0.986
Ave.	0.995	0.998	0.961	0.983	0.960	0.982	0.960	0.982

**Table 4:** Comparative analysis of performance metrics for action images generated from different scales of motion data.

	Accuracy			Precision			Recall			F1 Score		
	23 Joints	8 Joints	4 Joints	23 Joints	8 Joints	4 Joints	23 Joints	8 Joints	4 Joints	23 Joints	8 Joints	4 Joints
A1	0.999	0.999	0.999	0.980	0.993	0.986	1.000	0.993	1.000	0.990	0.993	0.993
A2	1.000	1.000	1.000	1.000	1.000	1.000	1.000	1.000	1.000	1.000	1.000	1.000
A3	0.993	0.992	0.991	0.921	0.937	0.913	0.965	0.931	0.944	0.942	0.934	0.928
A4	0.999	0.999	0.999	1.000	0.993	0.986	0.986	0.993	0.993	0.993	0.993	0.990
A5	0.999	1.000	0.997	1.000	0.993	0.979	0.979	1.000	0.979	0.989	0.997	0.979
A6	0.996	0.997	0.990	0.993	0.960	0.907	0.938	1.000	0.944	0.964	0.980	0.925
A7	1.000	1.000	0.998	0.993	0.993	0.986	1.000	1.000	0.986	0.997	0.997	0.986
A8	0.996	0.999	0.992	0.941	0.993	0.932	0.993	0.993	0.944	0.966	0.993	0.938
A9	0.997	0.996	0.996	0.979	0.972	0.986	0.979	0.965	0.944	0.979	0.969	0.965
A10	0.993	0.992	0.989	0.971	0.944	0.921	0.917	0.931	0.896	0.943	0.937	0.908
A11	0.999	0.998	0.995	0.993	1.000	0.965	0.986	0.972	0.958	0.990	0.986	0.962
A12	0.999	0.999	1.000	0.980	1.000	0.993	1.000	0.986	1.000	0.990	0.993	0.997
A13	0.999	0.998	0.997	0.993	0.979	0.993	0.993	0.993	0.951	0.993	0.986	0.972
A14	1.000	1.000	1.000	1.000	1.000	1.000	1.000	1.000	1.000	1.000	1.000	1.000
A15	0.999	0.996	0.998	0.986	0.965	0.986	0.993	0.965	0.986	0.990	0.965	0.986
A16	0.998	0.996	0.998	0.993	0.972	0.986	0.979	0.965	0.979	0.986	0.969	0.983
Ave.	0.998	0.998	0.996	0.983	0.981	0.970	0.982	0.980	0.969	0.982	0.981	0.969

**Table 5:** Performance comparison with the previous models.

Model	Accuracy	Precision	Recall	F1 Score
stgcn [41]	0.591	0.692	0.591	0.596
stgcn++ [42]	0.532	0.670	0.532	0.552
ctrgcn [43]	0.580	0.712	0.580	0.606
aagcn [44]	0.647	0.741	0.647	0.657
posec3d [19]	0.581	0.745	0.582	0.612
Multi-view 2D skeleton [45]	0.976	0.976	0.976	0.977
Proposed model	0.998	0.983	0.982	0.982

**Table 6:** Performance comparison under different levels of input errors.

Dataset	Accuracy	Precision	Recall	F1 Score
Without input errors	0.998	0.983	0.982	0.982
With 1% input errors	0.985	0.902	0.856	0.878
With 3% input errors	0.973	0.860	0.786	0.811

## Data Availability

Data cannot be provided due to the security reasons.
